# Full-Length cDNA Cloning, Molecular Characterization and Differential Expression Analysis of Lysophospholipase I from *Ovis aries*

**DOI:** 10.3390/ijms17081206

**Published:** 2016-07-28

**Authors:** Nan-Nan Liu, Zeng-Shan Liu, Pan Hu, Ying Zhang, Shi-Ying Lu, Yan-Song Li, Yong-Jie Yang, Dong-Song Zhang, Yu Zhou, Hong-Lin Ren

**Affiliations:** 1 Key Laboratory of Zoonosis Research, Ministry of Education/Institute of Zoonosis/College of Veterinary Medicine, College of Animal Sciences, Jilin University, Xi An Da Lu 5333, Changchun 130062, China; liunannan112117@163.com (N.-N.L.); zsliu1959@163.com (Z.-S.L.); hupan84@163.com (P.H.); zhangying201604@163.com (Y.Z.); lushiying1129@163.com (S.-Y.L.); l_ys92305@163.com (Y.-S.L.); zhouyu69@sina.com (Y.Z.); 2 Department of Food Science, College of Agriculture, Yanbian University, Yanji 133002, China; yjyang@ybu.edu.cn; 3 Animal Husbandry and Veterinary Unit of Xiangyang Town, Liuhe 135305, China; zhangdongsong2016@163.com

**Keywords:** Lysophospholipase I, *Ovis aries*, *Brucella*, tissue distribution, differential expression

## Abstract

Lysophospholipase I (LYPLA1) is an important protein with multiple functions. In this study, the full-length cDNA of the *LYPLA1* gene from *Ovis aries* (*OaLypla1*) was cloned using primers and rapid amplification of cDNA ends (RACE) technology. The full-length *OaLypla1* was 2457 bp with a 5′-untranslated region (UTR) of 24 bp, a 3′-UTR of 1740 bp with a poly (A) tail, and an open reading frame (ORF) of 693 bp encoding a protein of 230 amino acid residues with a predicted molecular weight of 24,625.78 Da. Phylogenetic analysis showed that the OaLypla1 protein shared a high amino acid identity with LYPLA1 of *Bos taurus*. The recombinant OaLypla1 protein was expressed and purified, and its phospholipase activity was identified. Monoclonal antibodies (mAb) against OaLypla1 that bound native OaLypla1 were generated. Real-time PCR analysis revealed that *OaLypla1* was constitutively expressed in the liver, spleen, lung, kidney, and white blood cells of sheep, with the highest level in the kidney. Additionally, the mRNA levels of *OaLypla1* in the buffy coats of sheep challenged with virulent or avirulent *Brucella* strains were down-regulated compared to untreated sheep. The results suggest that *OaLypla1* may have an important physiological role in the host response to bacteria. The function of *OaLypla1* in the host response to bacterial infection requires further study in the future.

## 1. Introduction

Lysophospholipase I (LYPLA1), also known as acyl-protein thioesterase 1 (APT1), is a widely distributed enzyme with phospholipase A2 [1], lysophospholipase [1,2] and acyl-protein thioesterase [3,4,5] activity. Phospholipids are important components in cellular membranes and are involved in signal transduction, mediator production and eicosanoid formation in both normal and disease states [6]. Lysophospholipids (LPLs) are important molecules in phospholipid metabolism and have received much attention as they are critical for cell survival and function [6,7,8]. LPLs are bioactive second messengers that modulate gene expression and are involved in multiple processes, such as stimulation of growth, phagocytosis of macrophages [6,9,10,11,12], activation of T lymphocytes [13,14], activation of many immune-related proteins [15], and promotion of anti-tumor and bactericidal activities [6]. LPLs are strictly regulated because increased levels of lysophospholipids are associated with many diseases [6,16]. The lysophospholipases (LYPLAs) are considered to be safeguards to ensure that normal levels lysophospholipids are maintained [6]. Multiple enzymes display LYPLA activity, and LYPLA1 belongs to the family of LYPLAs [4,16].

Palmitoylation involves the attachment of a 16-carbon fatty acid palmitate via a thioester bond to specific cysteine residues of target proteins and is an important post-translational modification that is critical for protein localization and function [5]. In contrast to other lipid modifications, such as isoprenylation and myristoylation, palmitoylation is a unique reversible post-translational modification [17] that allows proteins to rapidly shuttle between intracellular membrane compartments [18,19] and can be dynamically regulated by specific extracellular stimuli [18]. This modification is important for regulating protein subcellular localization, stability, trafficking, translocation to lipid rafts, aggregation, and interaction with effectors and other protein functions [17]. Recent studies have shown that palmitoylation is involved in endocytosis, reproduction, cell growth, fat and sugar homeostasis and signal transduction at the synapse [18]. Palmitoylation/depalmitoylation cycles are potential novel regulatory networks [20]. The multiple functions of palmitoylation suggest that it should be studied in detail. Palmitoylation is catalyzed by palmitoylation acyltransferases (PATs) while only a few enzymes catalyze depalmitoylation reactions [5,21].

LYPLA1 was identified as an acyl-protein thioesterase (APT), which is a depalmitoylation enzyme, in addition to palmitoyl protein thioesterases (PPT) [3], and can catalyze the removal of mislocalized palmitoylated proteins from endomembranes by depalmitoylation [21,22,23]. The PPT is localized predominantly in the lysosome [24]. However, LYPLA1 is principally localized in the cytosol, while some is present in the plasma membrane, the nuclear membrane and the endoplasmic reticulum (ER) [21].

Brucellosis is a zoonotic disease found worldwide that is caused by *Brucella*, resulting in infectious abortion and fever [25]. *Brucella* infections are chronic, and the interaction between the host and the *Brucella* pathogen is continuous [26]. Virulent *Brucella* strains invade the macrophages through lipid rafts and then reside in an acidified compartment, which fuses with components of the early endosomal pathway [27,28]. The macrophages kill the majority of *Brucella* cells at an early stage of infection [28,29], and the remaining *Brucella* cells establish and maintain a persistent intracellular infection in host cells using many virulence factors and strategies. *Brucella* can also induce the loss of the robust antigen-processing capacity of professional phagocytes and prevent phagosome-lysosome fusion and programmed cell death of infected macrophages, favoring pathogen survival and replication [26,30,31,32,33]. The host transcriptional responses against infection by *Brucella* have been characterized in several studies [28,34,35]. It is crucial to understand the host response to *Brucella*. In our lab, a time course suppression subtractive hybridization (SSH) cDNA library of buffy coats from sheep (*Ovis aries*) infected with different virulent *Brucella* strains was constructed to analyze the modulation of transcriptional profiles of hosts exposed to *Brucella* infection, and the differentially transcribed genes were screened. Among these genes, a partial cDNA of *LYPLA1* (*OaLypla1*) containing a full-length 3′-UTR was found to show differential expression in buffy coats from different virulent *Brucella*-infected sheep. As a result, the *OaLypla1* gene was chosen as a target candidate gene to further study the response of the host to *Brucella* infection. LYPLA1 is a protease with diverse biological functions that catalyzes multiple different reactions. It was reported that *LYPLA1* is down-regulated in macrophages after LPS stimulation [36]. However, the expression profiles of *LYPLA1* gene of the host infected with bacteria and whether *LYPLA1* takes part in the host immune response after the bacterial infection had not been investigated before. Thus, more information about the *OaLypla1* is required to better understand the potential relationship between the expression profiles of *OaLypla1* gene and bacteria infection. In this study, we identified the full-length cDNA sequence of a novel *LYPLA1* gene from *Ovis aries* (*O. aries*) for the first time. The recombinant OaLypla1 protein was expressed and purified, and its phospholipase activity was assessed. The tissue distribution of *OaLypla1* was determined, and differential expression profiles of *OaLypla1* in the buffy coats of sheep following challenge with different virulent *Brucella* strains were observed. Furthermore, we generated a monoclonal antibody (mAb) that reacts with the native OaLypla1. The results from this study may facilitate further study of the functions of *OaLypla1* in the host response to infection with *Brucella*.

## 2. Results

### 2.1. Characterization of OaLypla1 cDNA

**Figure 1:**
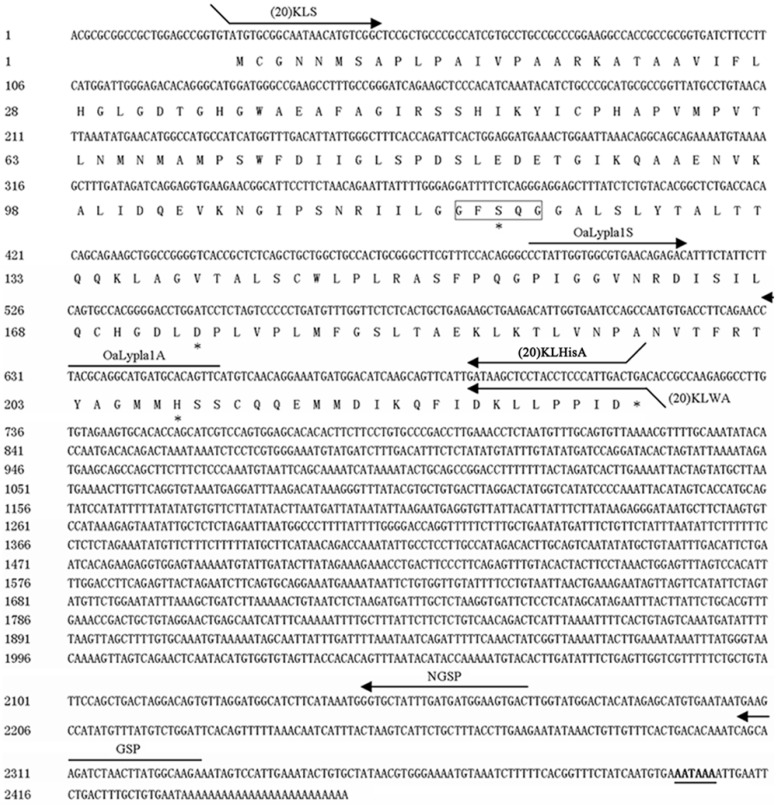
The full-length cDNA and the corresponding amino acid sequence of *OaLypla1*. The polyadenylation signal sequence (AATAAA) is shown in bold and underlined. The lipase motif GXSXG is boxed, and the catalytic triad is marked by asterisks (*). The arrows represent the places and lengths of the primers (5’-3’) used in this study.

The full-length cDNA sequence of the *OaLypla1* gene was obtained using 5′-RACE and deposited in GenBank (accession number KJ000742). The full-length *OaLypla1* cDNA was 2457 bp with a 5′-UTR of 24 bp, an ORF of 693 bp and a 3′-UTR of 1740 bp with a poly (A) tail downstream of a polyadenylation signal (AATAAA). The full-length nucleotide sequence and the deduced amino acid sequence of *OaLypla1* cDNA are shown in Figure 1.

The predicted OaLypla1 protein consisted of 230 amino acid residues with a predicted molecular weight of 24,625.78 Da and a theoretical isoelectric point of 6.77. The deduced OaLypla1 protein contained a ^117^GFSQG^121^ amino acid sequence corresponding to the GXSXG motif, which was located in an identical position to the amino acid sequence of the human LYPLA1 protein. A search using the BLASTn program in NCBI showed that the *OaLypla1* cDNA had 96% identity with the *LYPLA1* cDNA from *Bos taurus* (GenBank accession number: BC105143). Using the BLASTP program, the deduced amino acid of *O. aries* OaLypla1 was shown to exhibit high homology with the LYPLA1 proteins of other species, such as *Bos taurus* (99% identity), *Pongo abelii* (95% identity), *Homo sapiens* (95% identity), *Macaca mulatta* (94% identity), *Cricetulus griseus* (93% identity), *Xenopus tropicalis* (82% identity), and *Dicentrarchus labrax* (78% identity).

**Figure 2:**
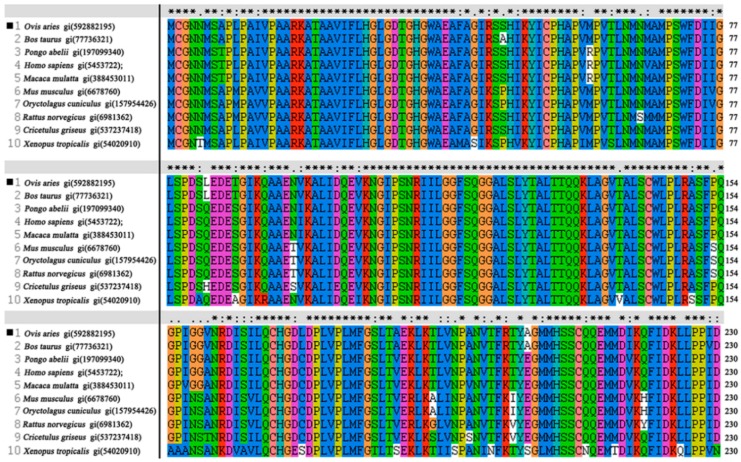
Multiple alignment analysis of the amino acid sequences of LYPLA1s from different vertebrates. The conserved amino acid residues of LYPLA1s are indicated by asterisks (*) above the column. Conserved substitutions are shown by colons (:), and dots (.) indicate semi-conserved amino acids. GenBank GI numbers of LYPLA1 protein sequences are given as follows: *Ovis aries* LYPLA1: gi (592882195); *Bos taurus* LYPLA1: gi (77736321); *Cricetulus griseus* LYPLA1: gi (537237418); *Homo sapiens* LYPLA1: gi (5453722); *Macaca mulatta* LYPLA1: gi(388453011); *Mus musculus* LYPLA1: gi (6678760); *Oryctolagus cuniculus* LYPLA1: gi (157954426); *Pongo abelii* LYPLA1: gi (197099340); *Rattus norvegicus* LYPLA1: gi (6981362); and *Xenopus (Silurana) tropicalis* LYPLA1: gi(54020910). The different colors are used to differ the proteinogenic 20 amino acids. Red: R and K; Brick red: G; Orange: C; Yellow: P; Green: N, S, T, and Q; Indigo: H and Y; Blue: M, A, L, I, V, F and W; Purple: D and E.

Multiple sequence alignment analysis of OaLypla1 was conducted using the known LYPLA1 proteins from several vertebrates to determine the level of amino acid conservation. The results showed that highly conserved acids were observed in the entire protein sequence as shown in Figure 2. The deduced amino acid sequence of LYPLA1 from *O. aries* had the Ser^119^, Asp^174^ and His^208^ triad that formed the catalytic site for LYPLA1 proteins from the mouse [37] and human [2]. All of these proteins shared the GXSXG motif sequence (^152^GFSQG^156^), which had a similar position and was found in the active site of serine proteases, esterases and lipases [6].

**Figure 3:**
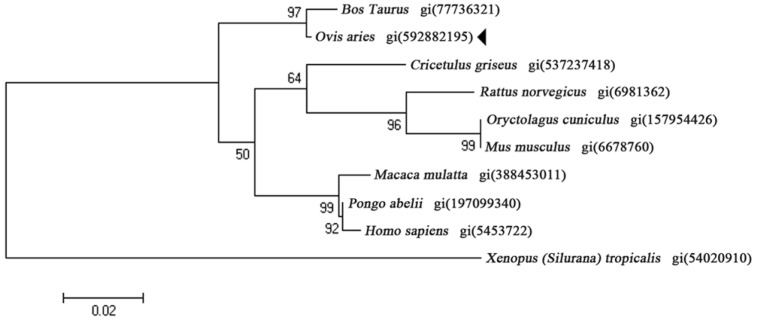
Phylogenetic relationship of LYPLA1 proteins from different species. The phylogenetic tree was constructed using MEGA 4.1 with the ClustalW algorithm. The number on the nodes indicates bootstrap values from 1000 replications.

To determine the phylogenetic relationships of OaLypla1, the amino acid sequences of LYPLA1 proteins from different species were selected. The phylogenetic tree was constructed by the neighbor joining method and revealed that the deduced amino acid sequence of OaLypla1 clustered with the ruminant subgroup near LYPLA1 from *Bos taurus* (Figure 3), suggesting that the gene cloned from *O. aries* belongs to the LYPLA1 family.

### 2.2. Protein Expression

**Figure 4:**
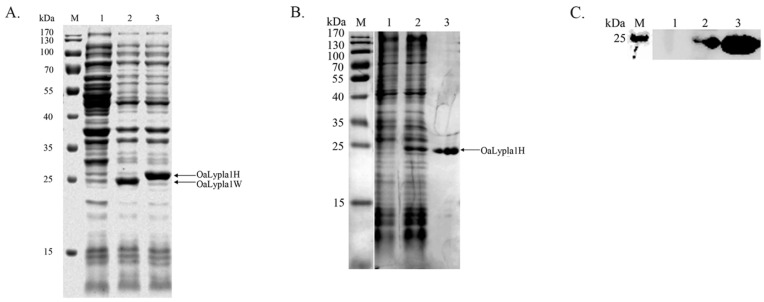
Expression and purification analysis of the recombinant OaLypla1 protein. (**A**) Expression analysis of the recombinant OaLypla1 by SDS-PAGE. M: protein marker (Thermo, Waltham, MA, USA); **lane 1**: total proteins from the uninduced pET-30a-LYPLA1-W cells; **lane 2**: total proteins from the induced pET-30a-LYPLA1-W cells (expressing OaLypla1 with no His_6_-tag); **lane 3**: total proteins from the induced pET-30a-LYPLA1-H cells (expressing OaLypla1 with a His_6_-tag) showing the shifted band due to the His_6_-tag; (**B**) Purification analysis of the recombinant OaLypla1H by SDS-PAGE. M: protein marker (Thermo, Waltham, MA, USA); **lane 1**: total proteins from the uninduced pET-30a-LYPLA1-H cells; **lane 2**: total proteins from the induced pET-30a-LYPLA1-H cells; **lane 3**: the purified recombinant protein OaLypla1H; (**C**) Expression and purification analysis of the recombinant OaLypla1H by Western blotting. M: protein marker (Thermo, Waltham, MA, USA); **lane 1**: total proteins from the uninduced pET-30a-LYPLA1-H cells; **lane 2**: total proteins from the induced pET-30a-LYPLA1-H cells; **lane 3**: the purified recombinant protein OaLypla1H.

The coding sequence of *OaLypla1* was cloned and inserted into the pET-30a vector. The recombinant proteins OaLypla1H (with a His_6_-tag) and OaLypla1W (with no tag) were over-expressed in *E. coli* BL21 (DE3). The results of SDS-PAGE analysis showed an approximately 25 kDa band in the induced cells, indicating that the recombinant OaLypla1H and OaLypla1W were successfully expressed in the transformed *E. coli* BL21 (DE3) cells following IPTG induction (Figure 4A). The recombinant OaLyplaH protein was purified and detected by SDS-PAGE and Western blots using a commercial His tag antibody and showed the same molecular weight of approximately 25 kDa, including the molecular weight of His_6_-tag (Figure 4B,C).

### 2.3. Activity Assay

**Figure 5:**
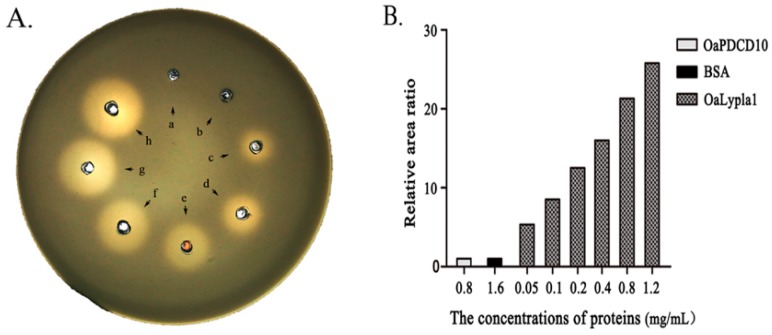
Phospholipase activity assay of the OaLypla1 protein. (**A**) Egg yolk/agarose diffusion test: a, 0.8 mg/mL OaPDCD10 with a His_6_-tag; b, 1.6 mg/mL BSA; and c–h, 0.05, 0.1, 0.2, 0.4, 0.8 and 1.2 mg/mL of OaLypla1H; (**B**) Relative area ratio. The results were calculated as a measure of the relative area ratio with the area of the hole in the center as 100%.

The phospholipase activity was determined by the egg yolk/agarose diffusion test. Phospholipase A is able to degrade micellar lecithins and cephalins into dissolvable lyso compounds and fatty acids, which leads to a clearing of egg yolk suspension in an agarose gel plate. Transparent rings will develop around the holes in which the phospholipase A is added after the egg yolk suspension is incorporated into the agarose gels [38]. The results of the egg yolk/agarose diffusion test showed that transparent rings were visualized around the holes after purified recombinant OaLypla1H was added. The diameters of the transparent rings were larger with increased concentrations of OaLypla1H. There were no transparent rings in the BSA and OaPDCD10 groups (Figure 5).

### 2.4. Specificity of the Monoclonal Antibody (mAb)

**Figure 6:**
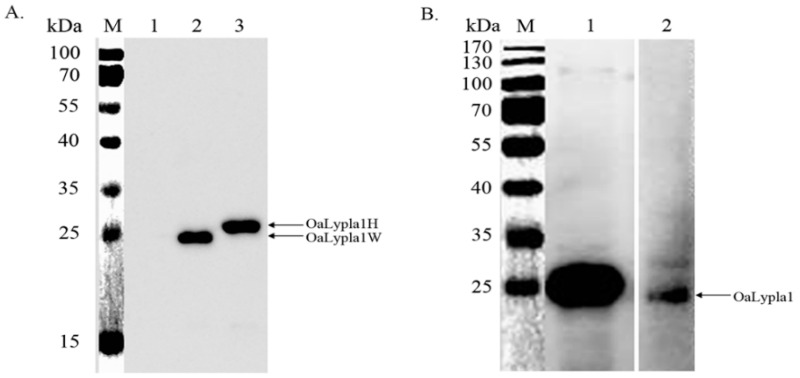
Immunoassay specificity of the monoclonal antibody against OaLypla1. (**A**) Immunoassay specificity against the recombinant OaLypla1. M: protein marker (Thermo, Waltham, MA, USA); **lane 1**: total proteins from the uninduced pET-30a-LYPLA1-W cells; **lane 2**: total proteins from the induced pET-30a-LYPLA1-W cells; **lane 3**: total proteins from the induced pET-30a-LYPLA1-H cells; (**B**) Immunoassay specificity against the native OaLypla1. M: protein marker (Thermo, Waltham, MA, USA); **lane 1**: total proteins from the induced pET-30a-LYPLA1-H cells as a positive control; **lane 2**: total proteins extracted from the kidney of *O. aries*. The pET-30a-LYPLA1-W cells were induced to express the recombinant OaLypla1 with no His_6_-tag, and the pET-30a-LYPLA1-H cells expressed OaLypla1 fused with a His_6_-tag.

A hybridoma cell line secreting mAbs against OaLypla1 was obtained as described in the methods and named OaLypla1-4A3. The results of the Western blotting analyses showed that the mAbs were able to bind the recombinant OaLypla1 and native OaLypla1 proteins extracted from kidney (Figure 6).

### 2.5. Tissue Distribution of OaLypla1

**Figure 7:**
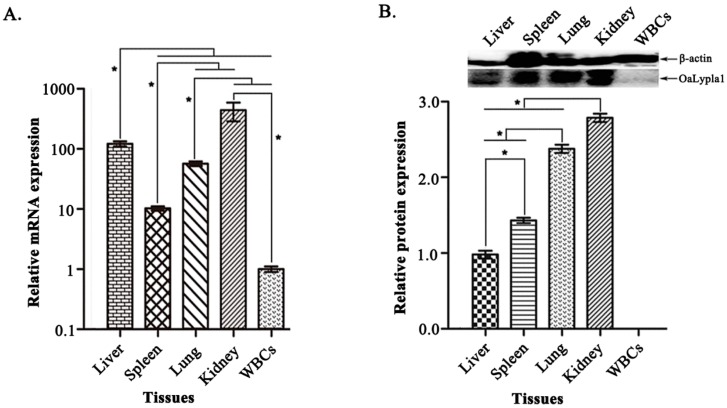
Tissue distribution of *OaLypla1*. (**A**) The tissue distribution of *OaLypla1* determined by quantitative real-time PCR. The mRNA levels in tissues were normalized with *β-actin*; (**B**) OaLypla1 protein detected in the tissues using Western blotting. Statistical differences among liver, spleen, lung, kidney, and white blood cells were determined by one-way analysis of variance (ANOVA) using SPSS 13.0 software (IBM, Armonk, NY, USA). Data are presented as the mean relative expression ± SD (*n* = 3). An asterisk indicates a statistically significant difference (* *p* < 0.05). WBCs: white blood cells.

To determine the tissue expression profiles of *OaLypla1*, qPCR and Western blot analyses were carried out to examine the tissue distribution of *OaLypla1* with the primers (Table 1) and the mAb prepared as described above. As shown in Figure 7A, the *OaLypla1* mRNA was detected in the liver, spleen, lung, kidney and WBCs, with the highest expression in the kidney. OaLypla1 protein was detected in the liver, spleen, lung and kidney but not in the WBCs (Figure 7B) by Western blotting.

### 2.6. OaLypla1 Expression Profiles after Challenge with Virulent and Avirulent Brucella Strains

**Figure 8:**
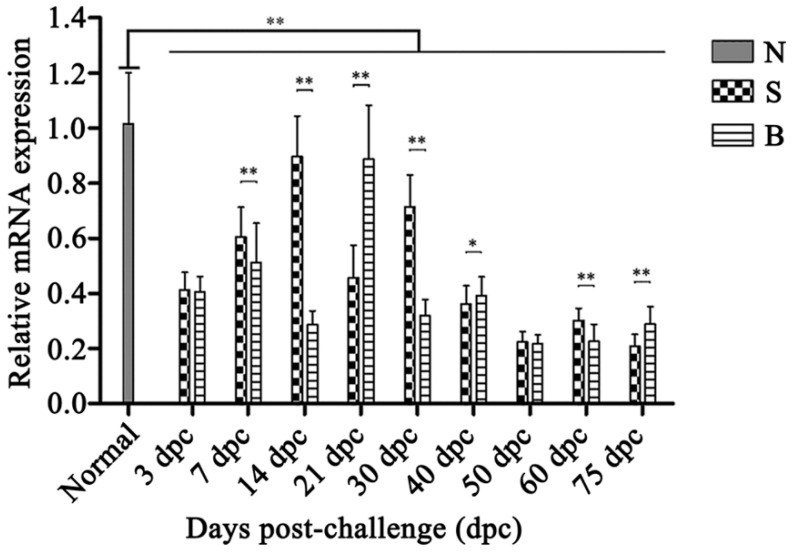
Differential expression of *OaLypla1* from buffy coats of *O. aries* challenged with different virulent *Brucella* strains. N: the normal sheep group; S: the group inoculated with the avirulent *Brucella suis* S2 strain; and B: the group challenged with the virulent field strain *Brucella melitensis*. Relative expression was calculated by the 2^−∆∆*C*t^ method using *O. aries β-actin* as an endogenous control. Statistical differences among the groups were determined by a one-way analysis of variance (ANOVA) using SPSS 13.0 software. Data are presented as the mean relative expression ± SD (*n* = 3) (* *p* < 0.05, ** *p* < 0.01).

The expression profiles of *OaLypla1* in the buffy coats of *O. aries* challenged with virulent and avirulent *Brucella* strains were analyzed using qPCR, as shown in Figure 8. Compared with the untreated control group, the transcription levels of *OaLypla1* were down-regulated in both the BmF-challenged group and the S2-challenged group from 3 to 75 dpc. In the S2-challenged group, the level of *OaLypla1* transcription increased from 3 to 14 dpc, reached a peak at 14 dpc, and then decreased, with the lowest level at 75 dpc. In the BmF-challenged group, the *OaLypla1* levels remained at a low level compared with the S2-challenged group. However, at 21 dpc, *OaLypla1* in the BmF-challenged group was much higher than in the S2-challenged group.

## 3. Discussion

Lysophospholipases are critical enzymes to regulate the multifunctional lysophospholipids. In this study, we focused on the *OaLypla1* gene screened from a time course SSH cDNA library constructed in our lab before. The full-length cDNA of *OaLypla1* was cloned and sequenced for the first time. The *OaLypla1* gene was characterized at the molecular level and the recombinant protein was produced to identify its functional activities in vitro. Further, the tissue-specific expression of *OaLypla1* was determined, and the bacterial stress responses of *OaLypla1* were investigated after sheep were infected with *Brucella*. This was the first report about the *LYPLA1* gene from sheep (*Ovis aries*).

It is reported that LYPLA1 of murine represents a member of the serine hydrolase family with Ser^119^, Asp^174^, and His^208^ composing the catalytic triad [39], and site-directed mutagenesis indicated that mutation of each residue to Ala abolished LYPLA1 activity [37,39]. The protein sequence analysis showed that OaLypla1 has the same catalytic triad with LYPLA1 from murine, rat and human. To confirm the bioactivity of the lipase catalytic center ^117^GFSQG^121^ in the OaLypla1 protein, the phospholipase activity of the recombinant OaLypla1 was identified using the egg yolk/agarose diffusion test in this study. This is a sensitive and simple test that has been used frequently for phospholipase A activity assay in recent years [40,41,42,43,44]. The purified recombinant OaLypla1 was also used to generate an anti-OaLypla1 mAb that was confirmed to specifically bind to the recombinant and native OaLypla1 proteins.

The tissue distribution of the *OaLypla1* gene at both the mRNA and protein levels was measured using qPCR and Western blots, respectively. *OaLypla1* was detected in all of the examined tissues, showing the highest mRNA expression in the kidney. The result was similar to the previous reports [2,4]. *OaLypla1* was constitutively expressed in tissues at the transcriptional level, suggesting that *OaLypla1* is a ubiquitously expressed gene and is a critical molecule that could potentially be involved in numerous physiological functions. OaLypla1 protein was not detected in WBCs, which was consistent with the low *OaLypla1* mRNA abundance in WBCs. As described in a previous study, gene expression levels cannot be determined only by mRNA abundance based on the mRNA–protein correlation [45], and post-transcriptional processing would also affect the expression levels of genes. The different mRNA and protein abundances resulting from the same gene emphasize the importance of integrative analysis of transcription and translation [46].

*LYPLA1* has been cloned from rat [4], murine [37], and human [2]. The lysophospholipase, phospholipase A2 and thioesterase activities were identified [21]. However, there is no investigation about the relationship between *LYPLA1* and infection of bacteria. The *LYPLA1* gene from *Ovis aries* cloned in this study may take apart in the immune response of the host towards the infection of bacteria.

Brucellosis is a major zoonotic disease worldwide. *Brucella* can evade the host immune response to survive and reproduce in host cells. Currently, live attenuated vaccines are used to eradicate brucellosis in cattle, sheep and goats. Infection by both the live attenuated vaccines and by the virulent strain will cause similar immune responses, such as stimulation of Th1 responses [47]. As a consequence, it is impossible to distinguish between vaccinated animals and infected ones using the available serological tests at present [48].

It was reported that the expression profiles and releases of *LYPLA1* are related to immunological stimulation and decreased *LYPLA1* levels likely contribute to macrophage responses to pro-inflammatory stimuli. Along with the mRNA and protein levels of *LYPLA1* decreased in LPS-stimulated RAW 264.7 cells, LYPLA1 released from RAW 264.7 cells into the culture medium was significantly increased. Inhibition of circulating LYPLA1 activity may be an effective treatment strategy for inflammation [36]. In the present study, the expression profiles of *OaLypla1* were analyzed using qPCR, and the results confirmed that the transcriptional levels of *OaLypla1* in WBCs were down-regulated by infection of *Brucella* compared with the uninfected sheep. Down-regulation of *OaLypla1* expression in WBCs from the infected groups showed that *LYPLA1* was perhaps involved in the immune response to the stimulation, and indicated that the transcription of the *LYPLA1* gene is inducible, which is consistent with the previous report [36]. There are hardly any studies about the relationship between the expression profile of *LYPLA1* and bacterial infection. LYPLA1 can hydrolyze lysophospholipids and participate in the phosphatidylcholine (PLC) pathway [39,49]. Thus, we speculated that the decreased LYPLA1 might result in the increasing level of LPLs, which are able to attract and activate macrophages and T or B cells, influence their interactions with other types of cells, and promote and modulate immune responses [50]. The exact mechanism of this phenomenon needs to be further studied. In addition, we also determined that the transcription patterns of the *OaLypla1* gene in buffy coats were different between sheep infected with the avirulent *Brucella* *suis* S2 vaccine strain and those infected with the virulent *Brucella* *melitensis* field strain. Whether *OaLypla1* can be a biomarker distinguishing between virulent *Brucella* infection and the avirulent *Brucella* vaccine inoculation and the relationship between *OaLypla1* and brucellosis need to be further studied in the future.

## 4. Materials and Methods

### 4.1. Animals and Cells

All of the sheep used in this research were purchased from Sangang farm (Jilin Province, China) and had no infectious diseases. Female BALB/c mice and sheep received food and water ad libitum [51]. All the animal experiments were carried out abiding by the provisions of EU animal management practices (24 November 1986), and approved by the Animals Ethics Committee of Jilin University of China in accordance with the Jilin university ethnic committee guideline for the Care and Use of Laboratory Animal (No. SCXK 2015-0004, 7 January 2015). The avirulent strain of *Brucella* (S2) was purchased from Harbin Pharmaceutical Group Bioengineering Co., Ltd. (Haerbin, China). The isolated and identified virulent strain of *Brucella*, myeloma SP2/0 cells, *Escherichia coli* DH5α competent cells and *E. coli* BL21 (DE3) cells were provided by the Key Laboratory of Zoonosis Research, Ministry of Education, Jilin University (Jilin, China).

### 4.2. Total RNA Isolation

Total RNA was isolated as described previously [48]. Briefly, the anticoagulant-containing blood collected from *O. aries* was centrifuged at 800× *g* for 15 min at 4 °C to extract the buffy coats. The buffy coats were used for total RNA extraction using TRIzol Reagent (Invitrogen, Carlsbad, CA, USA) following the manufacturer’s instructions. DNA and protein contamination was removed using recombinant DNase I (RNase-free) (TaKaRa, Dalian, China) and an RNeasy MinElute Cleanup Kit (Qiagen, Hilden, Germany).

### 4.3. 5′-RACE and Sequence Assembly

To obtain the full-length cDNA sequence, the purified total RNA and a SMARTer™ RACE cDNA Amplification Kit (Clontech, Mountain View, CA, USA) were used. A set of gene-specific primers was designed (Table 1) and named GSP (gene-specific primer) and NGSP (nested gene-specific primer). The PCR amplifications were performed using the following protocol. Briefly, 20 µL reaction volumes containing 5′-RACE-Ready cDNA as the template, primers (0.4 µL of 10 µM GSP and 2.0 µL UPM provided by the kit), 0.4 µL 10 mM dNTPs, and 0.4 µL 50× Advantage Polymerase mix were prepared. The conditions were 94 °C for 3 min; 20 cycles of 30 s at 94 °C, 30 s at 54.1 °C, 3 min at 72 °C; then a final extension at 72 °C for 5 min. The products were diluted 1:50 and used as template cDNA for the nested PCR. The nested PCR amplification was similar to the first PCR amplification except for the primers and template. The PCR conditions were 3 min at 94 °C; 25 cycles of 30 s at 94 °C, 30 s at 61.1 °C, 3 min at 72 °C; then the last extension at 72 °C for 5 min. The PCR products were visualized on a 1% agarose gel stained with ethidium bromide. After purification and ligation into the pMD^TM^18-T vector (TaKaRa, Dalian, China), the target DNA products were transformed into *E. coli* DH5α competent cells and sequenced by Shanghai Sangon Biological Engineering Technology & Service Co., Ltd. (Shanghai, China). The obtained sequences and the partial cDNA sequence identified from the SSH cDNA library were assembled to obtain the full-length cDNA sequence of *OaLypla1*.

### 4.4. Sequence Verification and Analysis

A homology search for the assembled sequence of *OaLypla1* was performed using the BLAST search programs at NCBI [52]. The molecular weight of the putative protein was predicted using the Expert Protein Analysis System [53]. Characteristic domains or motifs were identified using the Motif scan program [54]. The amino acid sequences of LYPLA1 from various species were retrieved from NCBI and analyzed using ClustalW version 1.83. The phylogenetic tree was constructed based on the amino sequence alignment with the neighbor joining method from the MEGA version 4.1 program.

### 4.5. Cloning of OaLypla1

The open reading frame (ORF) of the *Oalylpla1* transcript was amplified using the forward primer (20)KLS with a *Nde*I recognition site and the reverse primers (20)KLHisA (for the 6× His tag fusion) and (20)KLWA (for no fusion) with a *Xho*I recognition site at their 5′-ends, as listed in Table 1. The recombinant plasmids constructed in 4.3 were used as the template with Ex Taq (TaKaRa, Dalian, China), and the PCR reactions were performed at 94 °C for 30 s; then at 94 °C for 30 s, 60 °C for 30 s, 72 °C for 60 s for 32 cycles; and the final extension at 72 °C for 10 min. The PCR products were separated, purified and ligated into the pMD^TM^19-T Simple vector and transformed into *E. coli* DH5α cells. The cells with recombinant plasmids were confirmed with PCR using M13 forward and reverse primers, and the plasmids were then sequenced.

### 4.6. Expression and Purification of the Recombinant OaLypla1 Protein

The recombinant plasmids were digested with *Nde*I and *Xho*I restriction enzymes, and the products were purified and ligated into the restriction enzyme-digested pET-30a vectors. The recombinant expression plasmids were transformed into *E. coli* BL21 (DE3), and the cells carrying the plasmid were named pET-30a-LYPLA1-H and pET-30a-LYPLA1-W. A single transformant colony was grown overnight in 5 mL Luria-Bertani (LB) medium containing 50 µg/mL kanamycin at 37 °C, and the protein expression of OaLypla1H (OaLypla1 with His_6_-tag) and OaLypla1W (OaLypla1 with no His_6_-tag) was separately induced by adding isopropyl β-D-1-thiogalactopyranoside (IPTG) at a final concentration of 1.5 mM at 37 °C for 6.5 h. Total protein lysates extracted from the induced pET-30a-LYPLA1-H and pET-30a-LYPLA1-W cells were analyzed using 12% sodium dodecyl sulfate-polyacrylamide gel electrophoresis (SDS-PAGE).

Protein expression of OaLypla1H in 200 mL fresh LB medium was induced by adding IPTG at a final concentration of 1.5 mM at 37 °C for 6.5 h. The induced pET-30a-LYPLA1-H cells were harvested and resuspended in binding buffer (20 mM sodium phosphate, 30 mM imidazole, 0.5 M NaCl, pH 7.4) [48]. The supernatant was collected by centrifugation at 12,000× *g* for 30 min at 4 °C after the resuspended cells were sonicated. Then, the supernatant was loaded onto a HisTrap^TM^ FF crude (GE Healthcare, Pittsburgh, PA, USA) resin, and after washed with binding buffer for 10 times of column volume, the OaLypla1H protein was eluted with elution buffer (20 mM sodium phosphate, 0.5 M NaCl, 0.5 M imidazole, pH 8.0). The purified protein was dialyzed using 0.01 M PBS (pH 8.0).

The purified protein OaLypla1H was run on 12% SDS-PAGE with a protein marker (Thermo, Waltham, MA, USA) and stained with Coomassie brilliant blue R250. The molecular mass and the purity of the purified protein were assessed. Western blotting was performed with a commercial anti-His tag antibody (Abcam, Cambridge, MA, USA) to demonstrate that the recombinant OaLypla1H protein had been expressed and purified.

### 4.7. Phospholipase Activity Assay

The phospholipase activity of the OaLypla1H protein was analyzed as described in previous reports [38,55,56,57]. Briefly, the egg yolk was diluted 1:4 with 0.85% NaCl, and the supernatant (Buffer A) was collected by centrifugation at 3500 rpm for 2 min. Agarose (0.6 g) was dissolved in 100 mL 0.05 M NaAc (pH 7.5) at 120 °C for 10 min. Buffer A (3 mL) and 0.01 M CaCl_2_ (1 mL) were added to the 0.6% agarose NaAc solution when the temperature cooled to 50 °C, and the solution was then poured into glass plates (Ф 150 mm), and a hole was punched after the agarose solidified. A 0.85% NaCl solution (50 µL) containing different concentrations of purified OaLypla1H (0.05–1.2 mg/mL) was added into the holes of the agarose plates, and 50 µL of 0.85% NaCl containing 1.6 mg/mL of the bovine serum albumin (BSA) and 0.8 mg/mL of the recombinant programmed cell death 10 of *Ovis aries* (OaPDCD10) with a 6× His tag [58] were applied as negative controls. The plates were incubated at 37 °C for 24 h. The diameters of visible transparent circles appearing as a result of the phospholipase activity of OaLypla1H were measured.

### 4.8. Preparation of the mAb against the OaLypla1 Protein

Eight- to ten-week-old female BALB/C mice were immunized in the footpad with 100 µg of OaLypla1H emulsified with an equal volume of complete Freund’s adjuvant (CFA, Sigma, St. Louis, MO, USA) or incomplete Freund’s adjuvant (IFA, Sigma, St. Louis, MO, USA) based on a previous report [59]. The mice were housed with adequate food and water. Four days after the fourth immunization, the spleen cells isolated from the immunized mice were fused with myeloma cells (SP2/0) at a ratio of 10:1 in the presence of PEG1000. Then, the fused cells were cultured in 96-well cell culture plates using 20% (*v*/*v*) FBS/HAT (Sigma, St. Louis, MO, USA) medium, which was changed once per 4 days. Two weeks later, the hybridoma cells secreting the anti-OaLypla1 mAb were screened and cloned by limiting dilution at least three times. The culture supernatant of the positive hybridoma cells was used for the next experimental study.

### 4.9. Specificity Analysis

The specificity of the mAb was analyzed using Western blotting as described in a previous report [48]. Untagged OaLypla1W, OaLypla1H with a His_6_-tag and whole proteins from sheep kidneys were used to confirm the specificity of the mAb binding by Western blotting. Briefly, protein samples were extracted from bacteria or sheep kidneys, separated with 12% SDS-PAGE, and probed with the anti-OaLypla1 mAb (dilution 1:20) after transfer to a PVDF membrane (Millipore, Billerica, MA, USA). Then, the horseradish peroxidase-labeled goat anti-mouse IgG (dilution 1:2000) was incubated with the PVDF membrane. The membrane detection was performed with a BeyoECL Plus kit (Beyotime, Shanghai, China) using the ECL detection system (DNR, Jerusalem, Israel).

### 4.10. Tissue Distribution

Tissue distribution of *OaLypla1* in the liver, spleen, lung, kidney and white blood cells (WBCs) of sheep was analyzed by quantitative real-time PCR (qPCR) and Western blotting. Total RNA was extracted from different tissues and organs of 3 healthy sheep using TRIzol Reagent (Invitrogen, Carlsbad, CA, USA) following the manufacturer’s instructions. One microgram of total RNA was used to synthesize cDNA using a PrimeScript^TM^ RT reagent kit with gDNA Eraser (Perfect Real Time) (TaKaRa, Dalian, China). The qPCR reaction was carried out in a total volume of 20 µL containing 10 µL of FastStart Universal SYBR Green Master (ROX) (Roche, Basel, Switzerland), 0.6 µL of the forward primer (10 µM), 0.6 µL of the reverse primer (10 µM), and 1.5 µL of 5-fold diluted cDNAs. The qPCR cycling protocol was 95 °C for 10 min and 40 cycles of 15 s at 95 °C, 62 °C for 35 s and 72 °C for 32 s. Samples were normalized with *β-actin*, and the relative transcription level of *OaLypla1* was calculated using the 2^−ΔΔ*C*t^ method. Each assay was repeated in triplicate.

The total proteins from different tissues (liver, spleen, lung, kidney and WBCs) were extracted using RIPA lysis buffer (Beyotime, Shanghai, China) with 10 µL PMSF according to the manufacturer’s instructions. The total protein concentration was measured by the Bradford method [60] using a Bradford protein assay kit (Bio-Rad, Hercules, CA, USA). Total proteins (125 µg) from different sheep tissues and organs were used for Western blot analysis with the generated anti-OaLypla1 mAb following a standard protocol [48], and β-actin was used as an internal control. Optical densities of OaLypla1 and β-actin were calculated using Quantity One software (Bio-Rad, Hercules, CA, USA). Significant differences were determined by one-way analysis of variance (ANOVA) using the SPSS 13.0 software (IBM, Armonk, NY, USA).

### 4.11. Relative Transcript Level Analysis of OaLypla1 in Buffy Coats after Challenge with Virulent and Avirulent Brucella Strains

For differential expression analysis of *OaLypla1*, qPCR was employed with a pair of gene-specific primers (OaLypla1S and OaLypla1A) listed in Table 1, and *β-actin* (GenBank accession number U39357) was used as an internal control [48]. As described in a previous study [61], 9 sheep were randomly divided into three groups (*n* = 3). Three sheep were challenged with a virulent *Brucella* *melitensis* field strain (BmF) as the BmF-challenged group, and three sheep were inoculated with an avirulent *Brucella* *suis* S2 vaccine strain (S2) as the S2-inoculated group. Each sheep was injected with a total dose of 2.2 × 10^9^ cfu of bacteria. In addition, three other sheep were treated with the same volume of sterile 0.85% NaCl as the normal control group. The buffy coat samples from three individuals in each group were obtained at 3, 7, 14, 21, 30, 40, 50, 60 and 75 days post-challenge (dpc). Extracting the total RNAs and performing the qPCR for *OaLypla1* differential expression analysis in buffy coats were performed as described in 4.10 above. Each assay was repeated in triplicate.

## 5. Conclusions

This study identified and characterized a novel full-length cDNA of the *LYPLA1* gene from *Ovis aries* (*OaLypla1*), and the corresponding protein was expressed, purified and characterized. *OaLypla1* was widely expressed in different tissues at both the mRNA and protein levels. qPCR analysis showed that the expression of *OaLypla1* in the white blood cells of *Ovis aries* was down-regulated after infected with *Brucella*. This study provides fundamental data for further investigations exploring the relationship between *Brucella* infection and the expression patterns of *OaLypla1* and the possible functions of *OaLypla1* during *Brucella* infection.

## Figures and Tables

**Table 1:** Primers used in this study.

Primer	Object	Sequence (5′-3′)
GSP	5′-RACE	5′-TCTTGCCATAAGTTAGATCTTGCTG-3′
NGSP	5′-RACE	5′-GTCACTTCCATCATCAAATAGCACC-3′
(20)KLS	ORF amplification	5′-CATATGTGCGGCAATAACATGTCGGC-3′
(20)KLHisA	ORF amplification	5′-CTCGAGGTCAATGGGAGGTAGGAGCTTATC-3′
(20)KLWA	ORF amplification	5′-CTCGAGTCAGTCAATGGGAGGTAGGAGCTTATC-3′
β-actin-S	qPCR	5′-CCCAAGGCCAACCGTGAGAAGATGA-3′
β-actin-A	qPCR	5′-CGAAGTCCAGGGCCACGTAGCAGAG-3′
OaLypla1S	qPCR	5′-CCTATTGGTGGCGTGAACAGAGAC-3′
OaLypla1A	qPCR	5′-GAACTGTGCATCATGCCTGCGTAG-3′

The letters marked by the single underline in the primer sequences stood for the restriction sites of *Nde* I (CATATG) and *Xho* I (CTCGAG).
